# Charge-Trapping-Enhanced Resistive Switching and Charge Storage in Silk Fibroin–TiO_2_ Composite Memristors

**DOI:** 10.3390/mi17080880

**Published:** 2026-07-24

**Authors:** Seungmin Song, JunHyeong Park, JungBeen Cho, Seyeon Tak, Taehun Kim, Kyungtaek Min, Sung-Nam Lee

**Affiliations:** Department of Semiconductor Engineering, Tech University of Korea, Siheung 15073, Republic of Korea

**Keywords:** silk fibroin, memristor, TiO_2_ nanoparticles, resistive switching, bio-compatible memory

## Abstract

Silk fibroin (SF) is a promising bio-compatible material for transient and bio-integrated memory devices; however, its relatively high leakage current and limited resistance state stability remain critical issues. In this study, Ag/SF–TiO_2_/Pt bio-memristors were fabricated using SF–TiO_2_ composite films with TiO_2_ nanoparticle concentrations of 0, 0.25, 0.5, and 1.0 wt%. SEM analysis showed that TiO_2_ incorporation increased particle aggregation while maintaining continuous film morphology. Optical analyses revealed that TiO_2_ nanoparticles reduced the apparent optical gap, enhanced sub-bandgap absorption, and suppressed photoluminescence intensity, indicating the formation of defect- and trap-related states. Electrical measurements demonstrated that TiO_2_ incorporation effectively reduced leakage current and stabilized the high-resistance state. The devices exhibited stable bipolar resistive switching within ±1 V, with enhanced I_on_/I_off_ ratios of approximately 10^4^–10^5^ after TiO_2_ addition. Endurance and retention measurements confirmed reliable switching over 100 cycles and stable resistance states up to 10^4^ s. Capacitance analysis further revealed resistance state-dependent charge storage behavior, with higher capacitance in the low-resistance state due to conductive filament formation and TiO_2_-assisted interfacial polarization. These results indicate that TiO_2_ nanoparticles effectively modulate charge trapping, leakage suppression, and memory stability in SF-based bio-memristors.

## 1. Introduction

Memristors have attracted considerable attention as next-generation non-volatile memory devices for data storage and bio-compatible memory applications [1]. Their resistive switching (RS) behavior enables the reversible modulation of resistance states depending on the history of the applied electrical bias. This memory-dependent resistance modulation is highly advantageous for non-volatile information storage and resistance state control [2,3]. In particular, memristors can retain their resistance states even after the external power is removed, making them promising candidates for low-power non-volatile memory devices [4,5]. Despite these advantages, further improvements in switching stability, operating voltage, leakage current suppression, and device reliability are still required for practical applications [6,7,8]. Recent studies have shown that material selection, defect engineering, and interfacial control are key to reliable, low-power RS devices [9,10,11]. Recently, increasing attention has been paid to eco-friendly, biodegradable, and bio-compatible materials for next-generation electronic devices [9]. Among these materials, silk fibroin (SF), a natural protein polymer extracted from silkworm cocoons, has emerged as a promising active material for bio-compatible memristors [10]. SF offers several advantages, including mechanical flexibility, biodegradability, optical transparency, solution processability, and excellent film-forming capability [11]. These properties make SF particularly attractive for bio-integrated electronics, implantable devices, wearable sensors, and transient memory systems. The electrical and structural properties of SF films can be controlled through solution-based processes such as spin coating, which enables simple and low-cost thin-film fabrication [12,13]. In addition, SF can be combined with inorganic nanoparticles to tailor its electrical properties and improve device performance [14]. However, pristine SF-based memristors often suffer from relatively high leakage current, limited charge-trapping capability, and insufficient resistance state stability, which can restrict the memory window and long-term reliability of the devices [15]. Therefore, introducing functional nanoparticles into the SF matrix is an effective strategy for improving the resistive switching characteristics of SF-based bio-memristors.

TiO_2_ nanoparticles are promising additives for SF-based memristors because of their chemical stability, insulating nature, high dielectric response, and ability to introduce defect- and interface-related charge-trapping sites [16]. Other transition metal oxides, such as ZnO, HfO_2_, Ta_2_O_5_, WO_3_, NiO, and CuO, may also act as functional additives because they can provide defect-related charge-trapping sites, oxygen vacancy-assisted transport, and interfacial polarization [17,18,19]. The phase structure of TiO_2_ strongly influences RS behavior by affecting oxygen vacancies, surface states, interfacial defects, and charge trapping [20,21]. In this study, rutile TiO_2_ nanoparticles were used as inorganic additives because of their chemical stability, insulating characteristics, and defect-/interface-related trap sites [22,23]. When TiO_2_ nanoparticles are incorporated into the SF matrix, they can act as charge-trapping centers and local carrier-blocking regions, thereby suppressing leakage current and stabilizing the high-resistance state (HRS) [24]. Furthermore, TiO_2_-related trap states and interfacial polarization can modulate conductive filament formation and rupture, which are closely related to SET/RESET behavior, resistance state stability, and memory window enhancement [24,25]. TiO_2_ particle size, dispersion, and synthesis route strongly affect composite memristor performance. Well-dispersed nanoscale TiO_2_ nanoparticles provide interfacial charge-trapping sites, while excessive aggregation can cause electric field concentration and device variability. Therefore, TiO_2_ synthesis methods are important for controlling crystallinity, particle size, oxygen vacancies, and surface chemistry [23,26]. In addition to resistive switching, the resistance state-dependent capacitance behavior of SF–TiO_2_ composite memristors is also important for understanding charge storage, interfacial polarization, and filament-assisted charge accumulation [17]. In this study, Ag/SF–TiO_2_/Pt bio-memristor devices were fabricated using SF–TiO_2_ composite films with TiO_2_ nanoparticle concentrations of 0, 0.25, 0.5, and 1.0 wt%. The TiO_2_ concentration was varied to investigate its influence on charge trapping, RS behavior, and charge storage characteristics in SF-based bio-memristors. Unlike previous polymer-based nanoparticle memristor studies [27,28], this work correlates TiO_2_ concentration-dependent morphology, optical defect states, leakage current suppression, RS reliability, and resistance state-dependent capacitance behavior in SF–TiO_2_ bio-memristors. The effects of TiO_2_ incorporation on film morphology, optical absorption, photoluminescence, leakage current, resistive switching, endurance, retention, and capacitance characteristics were systematically investigated. This work demonstrates that TiO_2_ nanoparticle incorporation into SF films improves resistive switching stability by suppressing leakage current and enhancing charge trapping, while also enabling resistance state-dependent charge storage behavior in Ag/SF–TiO_2_/Pt bio-memristors.

## 2. Materials and Methods

The SF solution used in this study was prepared from Bombyx mori silkworm cocoons. The cocoons were cut into small pieces and boiled in an aqueous Na_2_CO_3_ solution for 60 min to remove sericin. The degummed silk fibers were thoroughly rinsed several times with deionized water and dried under ambient conditions. Subsequently, the dried silk fibers were dissolved in a LiBr solution at 60 °C for 2 h to obtain a homogeneous SF solution. The resulting solution was dialyzed using a dialysis cassette for 48 h to remove residual salts and impurities. Finally, the dialyzed SF solution was centrifuged at 4000 rpm to remove undissolved residues, yielding a purified SF solution [1,5]. Prior to device fabrication, sapphire substrates were cleaned using a standard RCA cleaning process to remove organic and inorganic contaminants. A Pt bottom electrode was then deposited on the cleaned sapphire substrate. To further improve the surface cleanliness and enhance the adhesion of the silk-based active layer, the Pt surface was treated with O_2_ plasma. Titanium dioxide nanoparticles, TiO_2_ rutile nanopowder, with a particle size of <100 nm, dimensions of approximately 10 nm × 40 nm, a surface area of 50 m^2^/g, and a purity of 99.5% trace metals basis, were purchased from Sigma-Aldrich, Darmstadt, Germany (Product No. 637262-25G). Silk–TiO_2_ composite solutions were prepared by adding TiO_2_ nanoparticles to the purified SF solution at concentrations of 0.25, 0.5, and 1.0 wt%. The mixed solutions were magnetically stirred for approximately 2 h to improve nanoparticle dispersion and then ultrasonicated for approximately 2 h to obtain more uniformly dispersed Silk–TiO_2_ composite solutions. The resulting Silk–TiO_2_ composite solutions, including the pristine SF solution as a reference sample, were spin-coated on the Pt bottom electrode at 5000 rpm for 30 s. After spin coating, the films were treated with methanol to induce structural stabilization of the SF matrix. The resulting Silk–TiO_2_ composite layer had an estimated thickness of approximately 1.0 µm, as determined by cross-sectional SEM analysis. Subsequently, Ag top electrodes were deposited on the Silk–TiO_2_ composite layer to complete the Ag/SF–TiO_2_/Pt memristor structure.

The surface morphology of SF-based thin films and fabricated devices was examined using optical microscope (OM) and scanning electron microscopy (SEM; COXEM, Daejeon, Republic of Korea). The optical properties of silk–TiO_2_ composite films were investigated using photoluminescence (PL) spectroscopy (Dongwoo, Seoul, Republic of Korea) with a 266 nm excitation laser. The optical absorption characteristics were analyzed using UV-visible spectroscopy (Thermo Fisher Scientific, Evolution 300, Waltham, MA, USA). The electrical characteristics of the Ag/SF–TiO_2_/Pt memristor devices were measured using a semiconductor parameter analyzer (Keithley 4200, Keithley Instruments, Cleveland, OH, USA). Bipolar voltage sweeps were applied to investigate the resistive switching behavior, including the SET and RESET processes. The high-resistance state (HRS) and low-resistance state (LRS) were extracted to evaluate the resistive switching performance. In addition, the log(I)–log(V) plots were analyzed to examine the dominant charge transport mechanisms in the silk–TiO_2_ composite layer. The charge storage behavior of the devices was investigated by measuring the capacitance–voltage (C–V) characteristics using an LCR meter/C–V meter (HP 4284A, Hewlett-Packard, Palo Alto, CA, USA). The C–V characteristics were measured by sweeping the applied voltage from −1.0 to +1.0 V. Frequency-dependent capacitance measurements were also performed over the kHz–MHz range to analyze the capacitive response, ion-related polarization behavior, and charge storage characteristics of silk–TiO_2_ composite films.

## 3. Results and Discussion

### 3.1. TiO_2_ Concentration-Dependent Particle Aggregation in SF–TiO_2_ Composite Films

**Figure 1:**
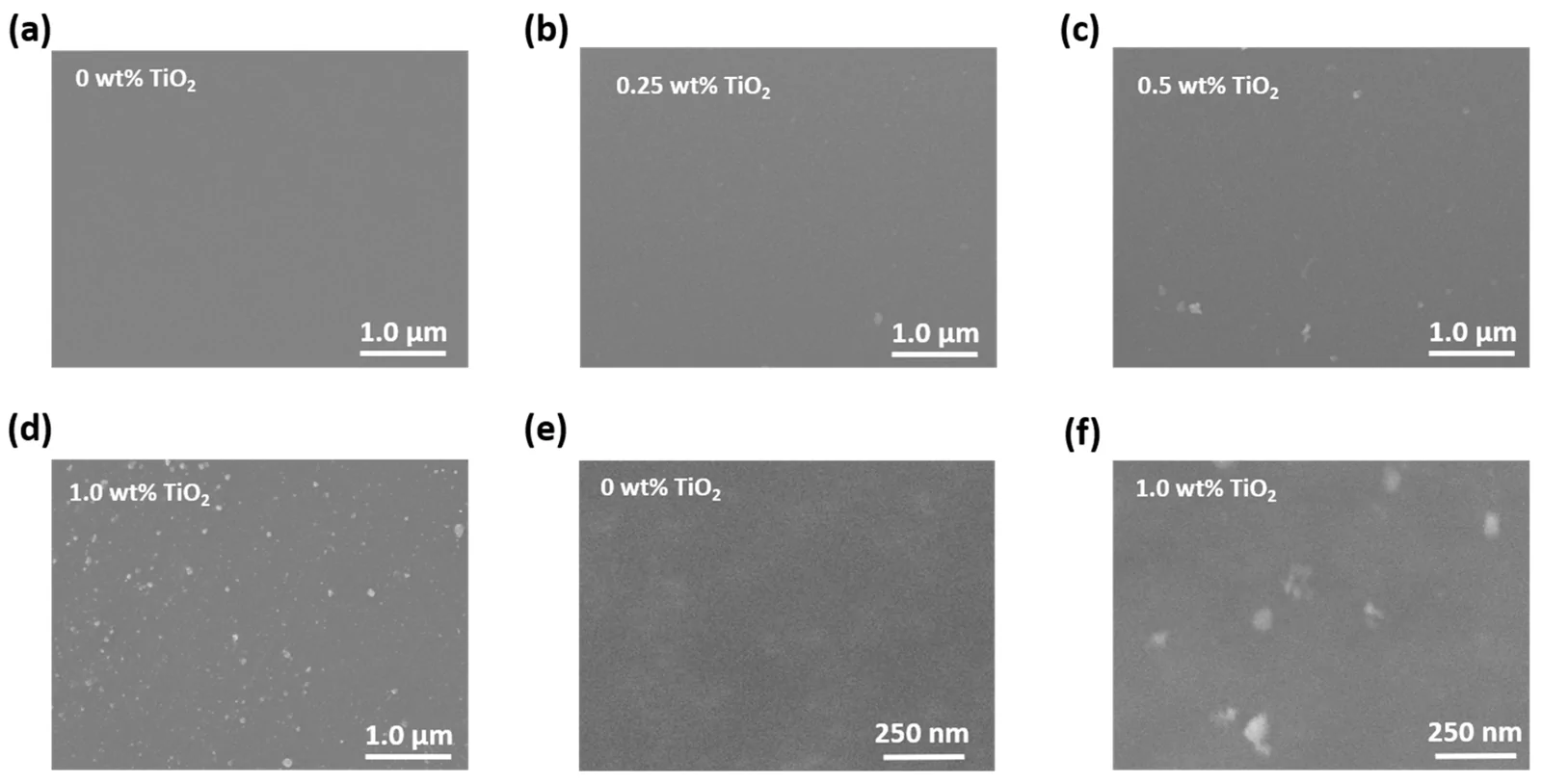
Surface SEM images of spin-coated SF-TiO_2_ composite films containing (**a**) 0, (**b**) 0.25, (**c**) 0.5, and (**d**) 1.0 wt% TiO_2_ nanoparticles, showing increased particle aggregation with increasing TiO_2_ concentration. Cross-sectional SEM images of the (**e**) pristine SF film and (**f**) 1.0 wt% TiO_2_-containing SF–TiO_2_ composite film.

Figure 1a–d show SEM images of the surface morphologies of spin-coated solid SF-TiO_2_ composite films with TiO_2_ nanoparticle concentrations of 0, 0.25, 0.5, and 1.0 wt%, respectively. All films were prepared by spin coating at 5000 rpm for 30 s, followed by drying at room temperature for at least 10 min to remove the residual solvent and stabilize the coated film. The pristine SF film in Figure 1a exhibits a relatively smooth and continuous surface without noticeable cracks or peeling, indicating the good film-forming ability of SF. After the incorporation of TiO_2_ nanoparticles, small granular features appear on the film surface, as shown in Figure 1b–d. In particular, the density and degree of particle aggregation gradually increase with increasing TiO_2_ concentration. At 0.25 wt% TiO_2_, the surface remains mostly uniform, with only slight nanoparticle-related roughness. As the TiO_2_ nanoparticle concentration increases to 0.5 wt%, more distinguishable particle clusters are observed. At 1.0 wt% TiO_2_, the aggregation of TiO_2_ nanoparticles becomes more pronounced, resulting in a rougher and more heterogeneous surface morphology. Figure 1e,f show cross-sectional SEM images of the pristine SF film and the 1.0 wt% TiO_2_-containing SF–TiO_2_ composite film, respectively. The pristine SF film shows a uniform cross-sectional structure, whereas TiO_2_ nanoparticles with an average size of approximately 50 nm are dispersed within the SF matrix in the 1.0 wt% composite film. These results indicate that TiO_2_ nanoparticles were successfully incorporated into the SF matrix; however, excessive TiO_2_ loading can promote nanoparticle agglomeration on the film surface. Nevertheless, no severe cracks, delamination, or discontinuities are observed even at the highest TiO_2_ concentration, suggesting that the SF–TiO_2_ composite films maintain sufficient mechanical integrity after spin coating and methanol treatment. The methanol treatment is expected to enhance the structural stability of the SF-based layer [29], thereby preventing serious film damage during the subsequent deposition of the Ag top electrode. The gradual increase in surface roughness and particle aggregation with TiO_2_ content can influence the electrical behavior of the Ag/SF–TiO_2_/Pt memristor devices. Moderate TiO_2_ incorporation may provide additional charge-trapping sites and facilitate resistive switching, whereas excessive aggregation at higher TiO_2_ concentrations may locally disturb the electric field distribution. Therefore, the TiO_2_ concentration plays an important role in controlling the morphology of the SF–TiO_2_ composite layer and, consequently, the resistive switching characteristics of the memristor devices.

### 3.2. TiO_2_ Concentration-Dependent Optical Properties of SF–TiO_2_ Composite Films

**Figure 2:**
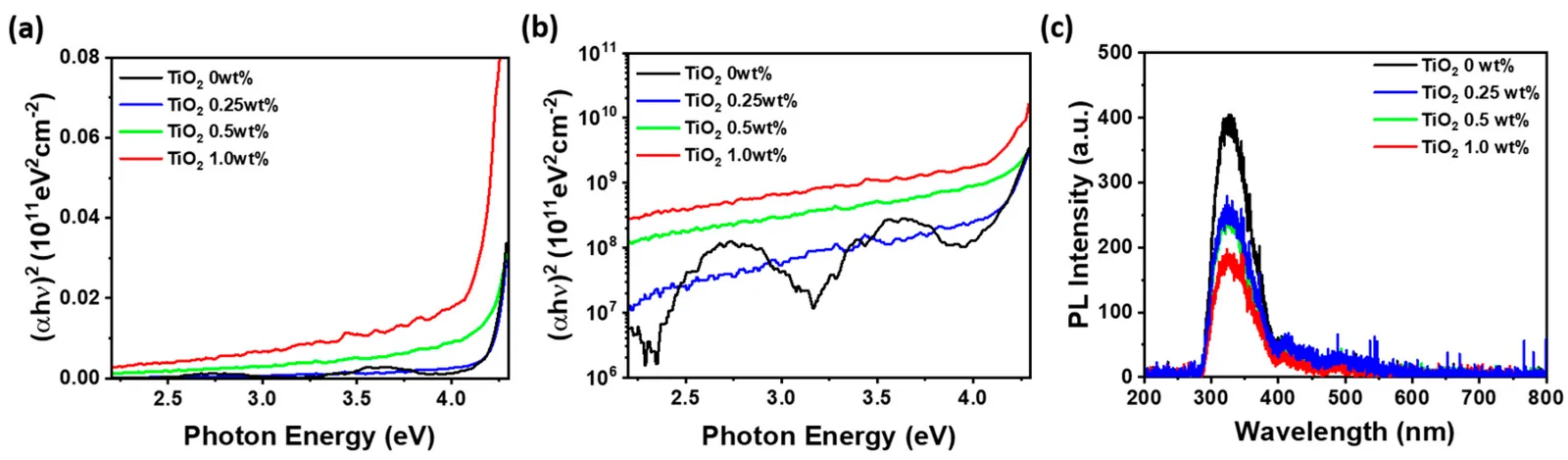
Optical properties of SF–TiO_2_ composite films with TiO_2_ concentrations of 0, 0.25, 0.5, and 1.0 wt%. (**a**) UV–visible absorption spectra and Tauc plots for estimating the apparent optical gap, (**b**) logarithmic absorption spectra showing enhanced sub-bandgap absorption tails, and (**c**) room-temperature PL spectra measured under 266 nm excitation.

Figure 2 shows the optical properties of SF-TiO_2_ composite films with TiO_2_ nanoparticle concentrations ranging from 0 to 1.0 wt%. Figure 2a presents the UV–visible absorption spectra and the corresponding Tauc plots used to estimate the apparent optical band gap of the SF-TiO_2_ film [17]. The pristine SF film exhibits relatively weak absorption in the UV region, whereas the absorption intensity gradually increases with increasing TiO_2_ concentration. This enhanced UV absorption is attributed to the incorporation of TiO_2_ nanoparticles into the SF matrix. From the Tauc plot analysis, the apparent optical band gap of the pristine SF film was estimated to be approximately 4.21 eV. With increasing TiO_2_ concentration, the band gap gradually decreased to 4.17, 4.12, and 4.09 eV for the SF–TiO_2_ composite films containing 0.25, 0.5, and 1.0 wt% TiO_2_, respectively. This gradual band gap narrowing suggests that TiO_2_ incorporation modifies the electronic structure of the SF matrix and introduces additional localized defect states within or near the band gap [30]. Figure 2b shows the absorption spectra plotted on a logarithmic scale to more clearly examine weak absorption features in the lower-energy region. Unlike Figure 2a, which focuses on the optical band gap estimated from the absorption edge, Figure 2b highlights the evolution of sub-bandgap absorption tails. As the TiO_2_ concentration increases, the absorption tail becomes more pronounced and extends toward the lower-energy region. This behavior indicates an increase in deep-level or trap-related absorption states in the SF–TiO_2_ composite films. These states may originate from TiO_2_-related defect levels, SF–TiO_2_ interfacial states, and surface states associated with nanoparticle aggregation [31,32]. The increase in deep-level absorption is consistent with the SEM results, where particle aggregation becomes more evident at higher TiO_2_ concentrations. Figure 2c shows the room-temperature PL spectra of the SF–TiO_2_ composite films measured under 266 nm excitation. A dominant emission peak is observed at approximately 330 nm, which can be attributed to the intrinsic emission of the SF-based matrix or near-band-edge-related recombination [33]. This peak represents the main radiative recombination process in the films. In contrast, only very weak emission features appear near 410 and 490 nm. These minor peaks are likely related to defect- or trap-assisted radiative recombination from localized states in the SF matrix, TiO_2_-related defects, or SF–TiO_2_ interfacial states [15,20]. As the TiO_2_ concentration increases, the overall PL intensity, especially the dominant 330 nm emission, gradually decreases. This PL quenching indicates that TiO_2_ nanoparticles introduce charge-trapping sites and non-radiative recombination pathways. It can be attributed to TiO_2_-induced localized defect states and SF/TiO_2_ interfacial trap states [34]. TiO_2_ nanoparticles can provide surface-related states and oxygen-vacancy-related defects, which capture photo-generated carriers and promote non-radiative recombination rather than radiative emission. Therefore, the decrease in the 330 nm emission intensity with increasing TiO_2_ concentration indicates enhanced carrier trapping and non-radiative recombination in the SF–TiO_2_ composite films. The slight changes in the weak 410 and 490 nm emissions further suggest that TiO_2_ incorporation modifies the defect-state distribution in the SF–TiO_2_ composite films. PLE measurements using a Xe lamp-based excitation source were attempted; however, reliable spectra could not be obtained because the SF–TiO_2_ composite films exhibit weak molecular/defect-related emission rather than strong band-to-band emission. The PL emission is mainly associated with intrinsic molecular fluorescence and localized electronic states in the SF matrix, while TiO_2_-related defect/interface states further promote non-radiative recombination. Therefore, the PL analysis was performed under fixed 266 nm laser excitation. Taken together, the Tauc plot, log-scale absorption, and PL analyses indicate that TiO_2_ incorporation narrows the apparent optical gap, enhances sub-bandgap absorption, and suppresses radiative recombination in the SF matrix. These optical results indicate that TiO_2_ incorporation modifies the electronic defect-state distribution in the SF matrix, as evidenced by the reduced apparent optical gap, enhanced sub-bandgap absorption, and PL quenching.

### 3.3. Electrical Characteristics of Ag/SF-TiO_2_/Pt Memristors: I–V Analysis

**Figure 3:**
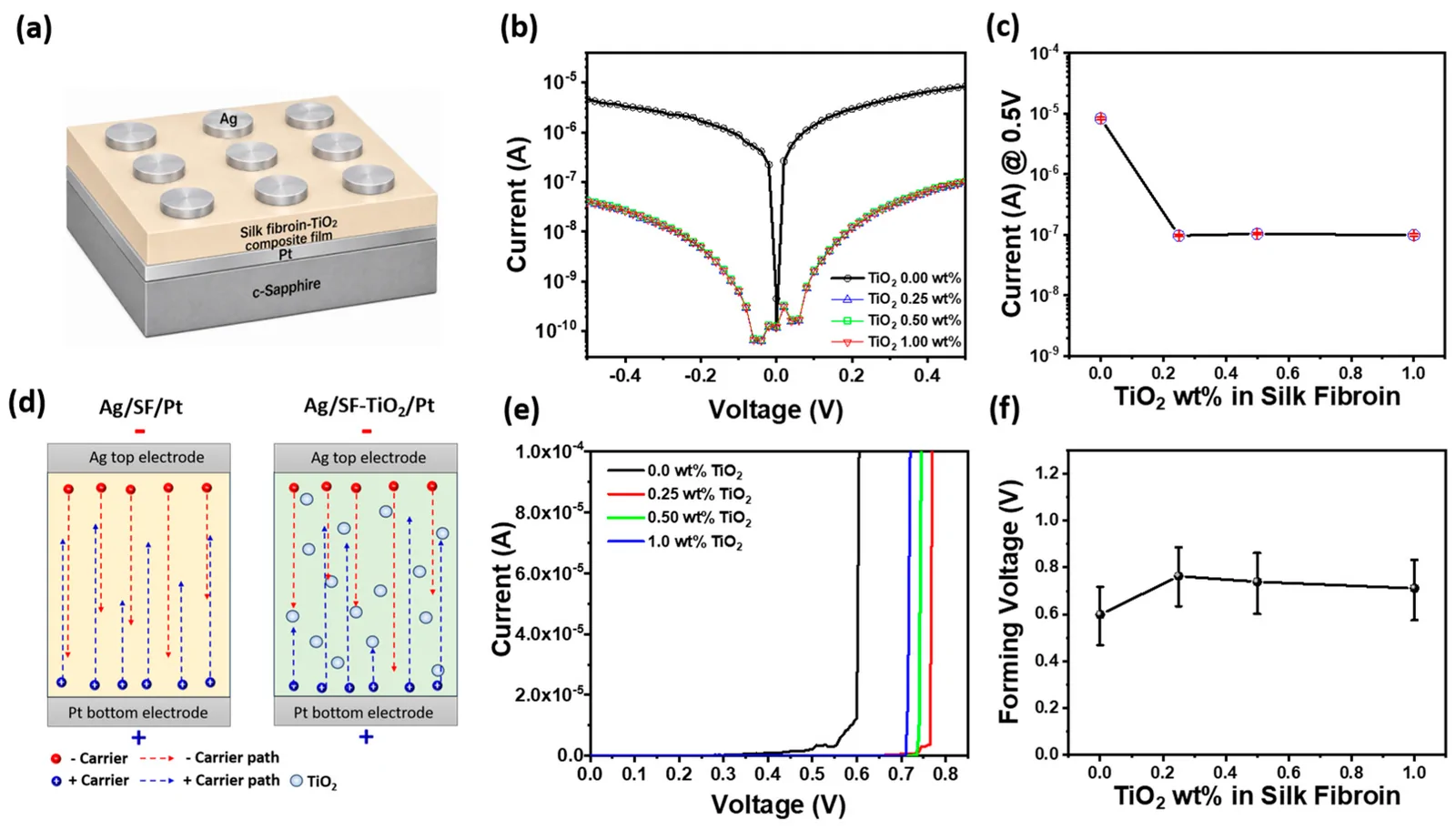
(**a**) Cross-sectional schematic of the Ag/SF–TiO_2_/Pt memristor on a c-sapphire substrate. (**b**) Initial I–V curves measured in the low-voltage range from −0.5 to +0.5 V. (**c**) Dependence of the current measured at +0.5 V on TiO_2_ concentration. (**d**) Schematic mechanism of leakage current suppression by TiO_2_ nanoparticles in the SF matrix. (**e**) Forming I–V characteristics of the devices with different TiO_2_ concentrations. (**f**) Variation in the forming voltage with TiO_2_ concentration. The error bars in (**c**,**f**) represent the standard deviations obtained from 10 devices fabricated on the same wafer.

Figure 3 shows the initial electrical characteristics and forming behavior of Ag/SF–TiO_2_/Pt memristor devices with TiO_2_ concentrations of 0, 0.25, 0.5, and 1.0 wt%. Figure 3a presents a schematic cross-sectional illustration of the Ag/SF–TiO_2_/Pt memristor fabricated on a c-sapphire substrate. The device consists of Ag top electrodes formed on the Silk–TiO_2_ composite film, a Pt bottom electrode, and a c-sapphire substrate. This schematic clearly illustrates the vertical metal–insulator–metal structure of the memristor and provides the structural basis for understanding the electrical characteristics discussed below. Figure 3b presents the I–V characteristics measured from −0.5 to +0.5 V to evaluate the initial leakage behavior of the SF–TiO_2_ composite films before pronounced conductive filament formation. The pristine SF device shows the highest current and nearly linear I–V behavior. In contrast, the current is significantly reduced after TiO_2_ incorporation and further decreases with increasing TiO_2_ concentration. Figure 3c shows the current values extracted at +0.5 V as a function of TiO_2_ content. The current decreases systematically as the TiO_2_ concentration increases, indicating that TiO_2_ nanoparticles suppress carrier transport in the SF matrix. This reduction can be attributed to the formation of TiO_2_-related charge-trapping sites and additional insulating barriers, which hinder leakage current in the initial state [21]. Therefore, TiO_2_ incorporation stabilizes the high-resistance state and is expected to improve the resistance contrast and switching stability of the Ag/SF–TiO_2_/Pt memristor devices. Figure 3d schematically illustrates the mechanism of leakage current modulation induced by TiO_2_ nanoparticles in the SF matrix. In the device without TiO_2_, carriers can move more directly through the SF film, resulting in easier carrier transport and relatively high leakage current. In contrast, when TiO_2_ nanoparticles are incorporated into the SF film, they introduce additional charge-trapping sites and interrupt or deflect the carrier transport paths [15,24]. As a result, carrier transport becomes more difficult, and the leakage current is effectively reduced. This schematic mechanism is consistent with the experimental results shown in Figure 3b,c, where the leakage current decreases with increasing TiO_2_ concentration. The leakage current suppression is related to the carrier-trapping and blocking effects of TiO_2_ nanoparticles in the SF matrix. In the pristine SF film, carriers can move through relatively continuous transport pathways. After TiO_2_ incorporation, however, carriers are captured at TiO_2_-related defect states and SF/TiO_2_ interfaces, resulting in interrupted transport paths and increased effective resistance. This explains the gradual decrease in leakage current with increasing TiO_2_ concentration [35]. Figure 3e presents the forming I–V characteristics of the Ag/SF–TiO_2_/Pt devices as a function of TiO_2_ concentration. All devices exhibit an abrupt increase in current during the positive voltage sweep, indicating the formation of conductive paths in the SF–TiO_2_ composite layer. Notably, the forming process occurs at relatively low voltages below 1.0 V for all TiO_2_ concentrations, suggesting that conductive filament formation can be initiated with a low electrical bias in the Ag/SF–TiO_2_/Pt structure. This low-voltage forming behavior may be related to the soft and ionically active nature of the SF matrix, as well as the involvement of Ag ion migration and TiO_2_-related trap-assisted conduction [24,36,37]. Figure 3f summarizes the forming voltage as a function of TiO_2_ concentration. The forming voltage increases from 0.599 V for the pristine SF device to 0.763 V for the device containing 0.25 wt% TiO_2_. This increase indicates that a small amount of TiO_2_ can suppress the initial formation of conductive paths by introducing charge-trapping sites and local insulating barriers. However, with further increasing TiO_2_ concentration, the forming voltage decreases from 0.763 V at 0.25 wt% to 0.711 V at 1.0 wt% TiO_2_. This non-monotonic behavior reflects the competing effects of two mechanisms: at low TiO_2_ content, insulating barriers dominate and raise the forming voltage, whereas at higher concentrations, nanoparticle aggregation intensifies local electric field enhancement and promotes trap-assisted Ag ion migration, thereby lowering the forming voltage [24,36]. Taken together, the results in Figure 3 indicate that the TiO_2_ concentration strongly affects both the leakage current and the forming behavior of the Ag/SF–TiO_2_/Pt memristor devices. TiO_2_ nanoparticles reduce the initial leakage current by introducing trap-assisted carrier blocking, while also exerting a non-monotonic influence on the forming voltage through the competing effects of insulating barrier formation and localized conductive path generation.

### 3.4. TiO_2_ Concentration-Dependent Resistive Switching Characteristics of Ag/SF–TiO_2_/Pt Memristors

**Figure 4:**
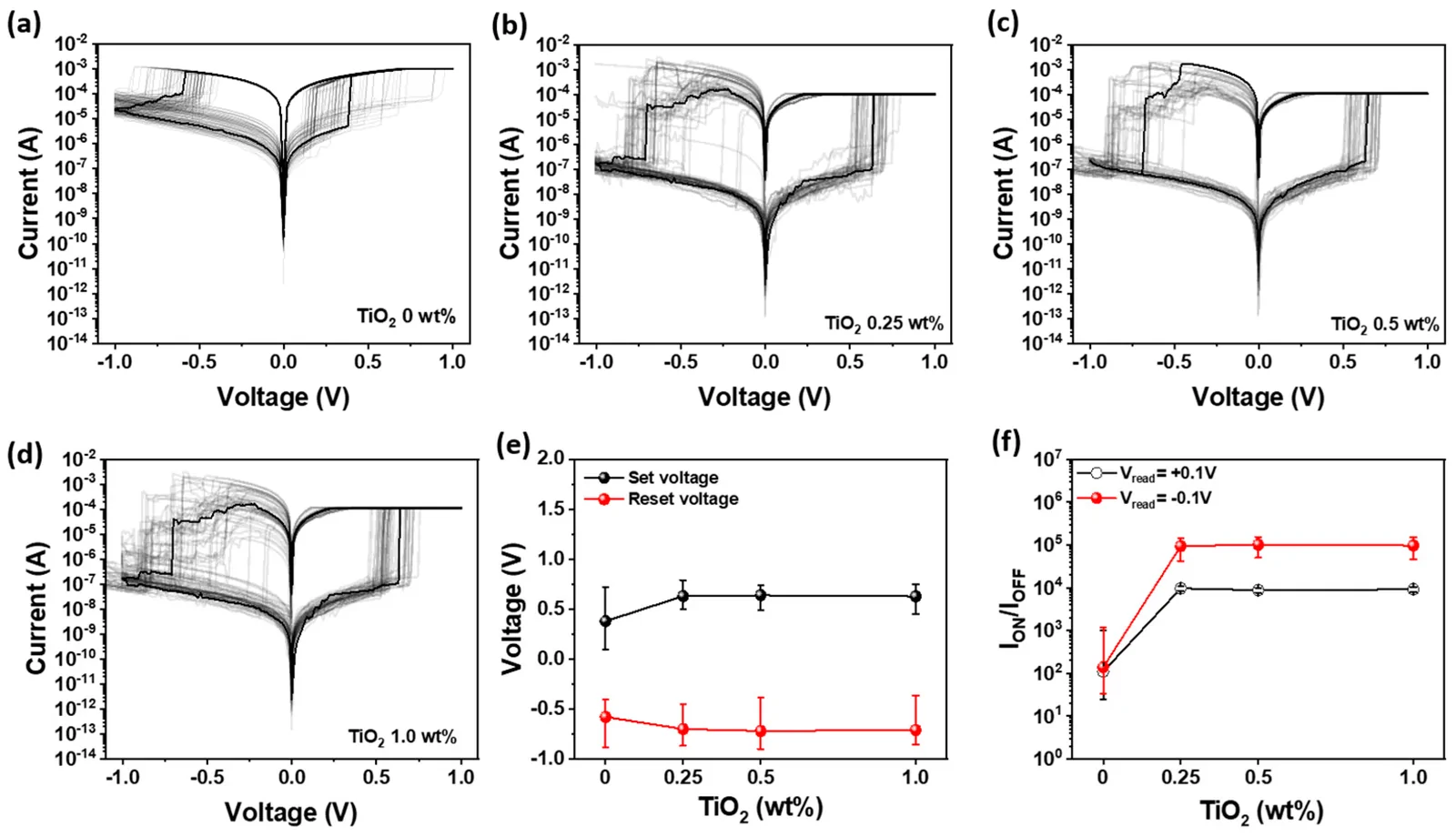
Resistive switching characteristics of Ag/SF–TiO_2_/Pt memristor devices with TiO_2_ concentrations of 0–1.0 wt%. (**a**–**d**) 100-cycle bipolar I–V curves for devices with (**a**) 0, (**b**) 0.25, (**c**) 0.5, and (**d**) 1.0 wt% TiO_2_. (**e**) Average set and reset voltages as a function of TiO_2_ concentration. (**f**) I_on_/I_off_ ratios measured at read voltages of +0.1 and −0.1 V, showing an enhanced memory window after TiO_2_ incorporation. The error bars in (**e**,**f**) represent the standard deviations calculated from 100 consecutive switching cycles.

Figure 4a–d show the resistive switching (RS) characteristics of Ag/SF-TiO_2_/Pt memristors with TiO_2_ concentrations of 0, 0.25, 0.5, and 1.0 wt%, respectively. The RS characteristics were repeatedly measured for 100 consecutive cycles to evaluate the switching stability and cycle-to-cycle variation in each device. All devices exhibit stable bipolar RS behavior within the range of ±1.0 V, indicating that conductive paths can be repeatedly formed and ruptured in the SF-based active layer. Although conductive filaments were not directly observed, the abrupt forming/SET transition, bipolar RESET behavior, and stable HRS/LRS separation are consistent with conductive path formation and rupture in the SF–TiO_2_ composite layer. The reduced leakage current and increased HRS resistance after TiO_2_ incorporation further suggest TiO_2_-assisted carrier trapping and blocking. Therefore, the proposed switching mechanism is described as an indirect but reasonable interpretation based on the electrical characteristics. The gray curves represent the individual switching cycles, while the black curves indicate the representative or averaged switching behavior. Compared with the pristine SF device in Figure 4a, the TiO_2_-containing devices in Figure 4b–d exhibit a more clearly separated high-resistance (HRS) and low-resistance state (LRS). In particular, TiO_2_ incorporation suppresses leakage current in the HRS, which improves the distinction between the ON and OFF states. Figure 4e shows the average SET and RESET voltages extracted from 100 RS cycles. The pristine SF device exhibits average SET and RESET voltages of approximately 0.38 V and −0.58 V, respectively. With the addition of 0.25 wt% TiO_2_, the average SET and RESET voltages increase to approximately 0.63 V and −0.7 V, respectively. This increase suggests that the TiO_2_ nanoparticles introduce charge-trapping sites and local insulating barriers in the SF matrix, thereby requiring a slightly higher electric field for conductive filament formation and rupture [24,38,39,40]. Above 0.25 wt% TiO_2_, the average SET and RESET voltages remain almost unchanged, demonstrating stable operating voltage in the TiO_2_-containing devices. 

Figure 4f shows the I_on_/I_off_ ratio, defined as the LRS/HRS current ratio, measured at read voltages of +0.1 and −0.1 V. The pristine SF device shows an I_on_/I_off_ ratio of approximately 10^2^, whereas the TiO_2_-containing devices exhibit a significantly enhanced I_on_/I_off_ ratio. In particular, for TiO_2_ concentrations of 0.25–1.0 wt%, the I_on_/I_off_ ratio reaches approximately 10^4^ at +0.1 V and 10^5^ at −0.1 V. This polarity-dependent asymmetry in the I_on_/I_off_ ratio is attributed to the directional nature of Ag ion migration in the Ag/SF–TiO_2_/Pt structure. Under negative read bias, the electric field drives Ag ions more effectively toward the Ag top electrode, promoting residual filament retention and enhancing the LRS conductance, while simultaneously reducing HRS leakage through stronger charge trapping at TiO_2_ sites. This results in a larger resistance contrast at −0.1 V compared with +0.1 V. This enhancement results from the reduction in HRS leakage current caused by TiO_2_-induced charge trapping and carrier blocking. Therefore, TiO_2_ incorporation effectively enlarges the memory window and improves the switching reliability of the Ag/SF–TiO_2_/Pt memristor devices.

### 3.5. Endurance and Retention Characteristics of Ag/SF–TiO_2_/Pt Memristors

**Figure 5:**
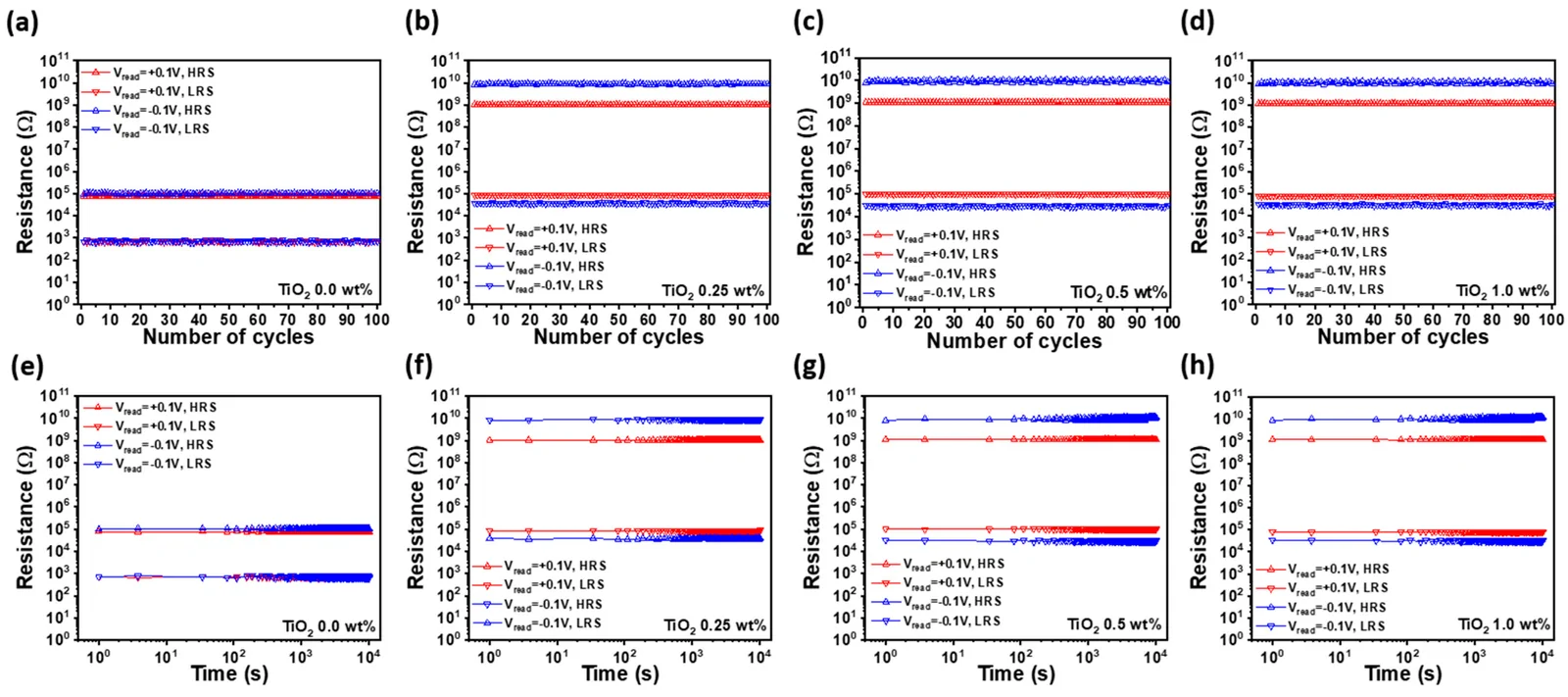
Endurance and retention characteristics of Ag/SF–TiO_2_/Pt memristor devices with different TiO_2_ concentrations. (**a**–**d**) Cycle-dependent HRS and LRS values extracted from the 100-cycle RS curves at read voltages of +0.1 and −0.1 V for devices containing (**a**) 0, (**b**) 0.25, (**c**) 0.5, and (**d**) 1.0 wt% TiO_2_. (**e**–**h**) Time-dependent HRS and LRS values measured up to 10^4^ s at read voltages of +0.1 and −0.1 V for devices containing (**e**) 0, (**f**) 0.25, (**g**) 0.5, and (**h**) 1.0 wt% TiO_2_.

To further verify the reliability of the Ag/SF-TiO_2_/Pt memristor devices, endurance and retention characteristics were investigated. Figure 5a–d show the endurance characteristics extracted from the 100-cycle RS curves in Figure 4a–d. The HRS and LRS values were monitored as a function of switching cycle at read voltages of +0.1 V and −0.1 V for devices containing Figure 5 (a) 0, (b) 0.25, (c) 0.5, and (d) 1.0 wt% TiO_2_, respectively. Regardless of the read voltage polarity, the HRS and LRS remain well separated and are maintained throughout the 100 switching cycles, demonstrating stable and reproducible resistive switching operation. With the incorporation of TiO_2_ nanoparticles, both HRS and LRS values increase compared with those of the pristine SF device. This result indicates that TiO_2_ nanoparticles suppress carrier transport in the SF matrix by introducing charge-trapping sites and local carrier-blocking barriers. In addition, when the TiO**_2_** concentration increases from 0.25 to 1.0 wt%, no significant change in either HRS or LRS is observed, suggesting that the resistance states become relatively saturated after TiO_2_ incorporation above 0.25 wt%. Figure 5e–h present the retention characteristics of the HRS and LRS measured as a function of time up to 10^4^ s at read voltages of +0.1 and −0.1 V for devices containing.

Figure 5 (e) 0, (f) 0.25, (g) 0.5, and (h) 1.0 wt% TiO_2_, respectively. Similar to the endurance results, the HRS and LRS values increase after TiO_2_ incorporation, indicating that TiO_2_ nanoparticles effectively reduce leakage current and enhance the resistance of both states. However, for TiO_2_ concentrations above 0.25 wt%, the resistance values do not change significantly, which is consistent with the cycle-dependent resistance behavior shown in Figure 5b–d. At all TiO_2_ concentrations, both HRS and LRS remain stable without noticeable degradation for 10^4^ s, confirming the non-volatile memory behavior of the devices. This stability indicates that the conductive state and charge-trapping configuration in the SF–TiO_2_ composite films are well preserved over time. These results show that adding TiO_2_ nanoparticles not only improves the initial switching performance but also enhances both cycle-to-cycle stability and long-term data retention by suppressing leakage current and stabilizing charge-trapping behavior. In conclusion, the Ag/SF–TiO_2_/Pt memristors exhibit reliable endurance over 100 switching cycles and stable retention over 10^4^ s, making them promising candidates for bio-compatible non-volatile memory devices.

### 3.6. Resistance State-Dependent Capacitance Characteristics of Ag/SF–TiO_2_/Pt Memristors

**Figure 6:**
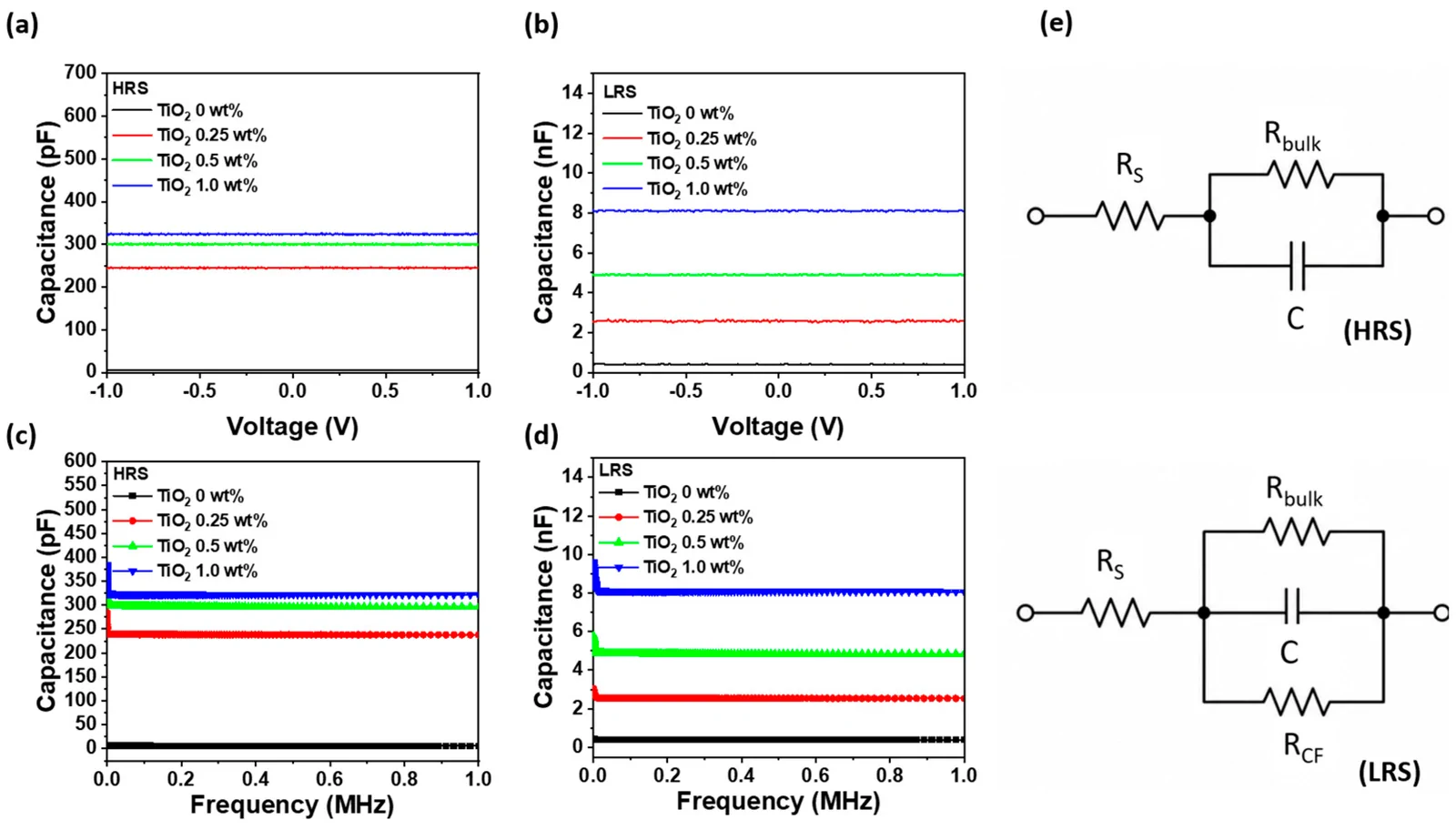
Capacitance characteristics of Ag/SF–TiO_2_/Pt memristor devices with different TiO_2_ concentrations. (**a**,**b**) Capacitance–voltage (C–V) characteristics measured from −1.0 to +1.0 V in the (**a**) HRS and (**b**) LRS. (**c**,**d**) Frequency-dependent capacitance characteristics measured up to 1 MHz in the (**c**) HRS and (**d**) LRS. (**e**) Equivalent-circuit models for the HRS and LRS. In the LRS, an additional conductive filament resistance (R_CF_) is introduced in parallel with the RC component.

Figure 6 shows the capacitance characteristics of Ag/SF–TiO_2_/Pt memristor devices with different TiO_2_ concentrations depending on the resistance state. Figure 6a,b present the capacitance–voltage (C–V) characteristics measured in the HRS and LRS, respectively. The measurements were performed over a voltage range from −1.0 to +1.0 V. The C–V measurements were performed using a small AC signal of 50 mV at 1 MHz, which limited slow ionic migration and conductive filament evolution; thus, the measured curves mainly reflect the small-signal capacitance response rather than intentional SET/RESET switching. In both resistance states, the capacitance remains nearly constant with increasing applied voltage, indicating stable capacitive behavior of the SF–TiO_2_ composite films under low-bias conditions. In the HRS, the capacitance gradually increases as the TiO_2_ concentration increases from 0 to 1.0 wt%, as shown in Figure 6a. This result suggests that TiO_2_ nanoparticles enhance the charge storage capability of the SF matrix by providing additional polarization centers and charge-trapping sites [17]. In the HRS, the physical origin of the capacitance enhancement is mainly related to TiO_2_-induced dipolar polarization and trap-assisted charge accumulation within the insulating SF matrix. The SF/TiO_2_ interfaces can serve as additional charge storage regions, leading to increased capacitance with increasing TiO_2_ concentration [21]. The increased capacitance in the HRS is consistent with the leakage current suppression and resistance window enhancement discussed in the previous sections. Figure 6b shows the C–V characteristics in the LRS. Compared with the HRS, the LRS exhibits much higher capacitance values, reaching the nF range. This large capacitance enhancement is attributed to the formation of conductive filaments in the SF–TiO_2_ composite layer. In the LRS, conductive paths can increase the effective charge storage region and strengthen interfacial polarization between the conductive filaments, TiO_2_ nanoparticles, and the SF matrix [41,42]. The much higher capacitance in the LRS can therefore be explained by filament-assisted interfacial charge accumulation. Once conductive filaments are formed, additional interfaces are created among the conductive paths, TiO_2_ nanoparticles, and the surrounding SF matrix, which increases the effective charge storage area and enhances interfacial polarization [41,42]. Therefore, the difference in capacitance between the HRS and LRS indicates that the resistance state of the device is closely coupled with its charge storage behavior. Figure 6c,d show the frequency-dependent capacitance characteristics measured in the HRS and LRS, respectively. In the HRS, the capacitance remains in the pF range and shows only a weak frequency dependence over the measured frequency range up to 1 MHz. The capacitance increases with increasing TiO_2_ concentration, indicating that TiO_2_ nanoparticles contribute to stable charge storage even under AC operation. In the LRS, the capacitance is significantly higher than that in the HRS and remains in the nF range. The LRS capacitance is relatively high at low frequencies and gradually decreases with increasing frequency, which is consistent with the typical dispersion behavior of interfacial polarization and ion-related charge accumulation. At low frequencies, slow polarization processes such as ionic redistribution and filament-assisted interfacial charge accumulation can follow the AC signal, contributing to the elevated capacitance. At higher frequencies, these slow polarization mechanisms can no longer respond to the rapidly alternating field, resulting in a gradual reduction in capacitance [41,42]. To further explain the resistance state-dependent capacitance behavior, an equivalent-circuit model is proposed, as shown in Figure 6e. In the HRS, the device is represented by the series resistance of the electrodes (R_s_) and a parallel combination of the bulk resistance (R_bulk_) and capacitance (C) of the insulating SF–TiO_2_ composite layer. Here, the capacitance is associated with TiO_2_-induced dipolar polarization, trap-assisted charge accumulation, and SF/TiO_2_ interfacial polarization. In the LRS, an additional low-resistance conductive filament path (R_CF_) is formed in parallel with the insulating matrix. The formation of this filamentary path creates additional interfaces between the conductive path and the surrounding SF–TiO_2_ matrix, thereby enhancing interfacial charge accumulation and increasing the effective capacitance. Therefore, the resistance-dependent capacitance can be attributed to the different charge storage configurations in the HRS and LRS. These results demonstrate that TiO_2_ incorporation enhances the capacitance of the SF-based memristor and that the capacitance is strongly dependent on the resistance state. The enhanced capacitance originates from TiO_2_-induced polarization, charge trapping, and conductive filament-assisted interfacial charge storage. Therefore, the Ag/SF–TiO_2_/Pt memristor exhibits coupled resistance and capacitance modulation, suggesting its potential for multifunctional bio-compatible memory devices. 

Furthermore, the optical defect states are closely correlated with the electrical performance of the Ag/SF–TiO_2_/Pt memristors. The enhanced sub-bandgap absorption and PL quenching observed in Figure 2 suggest the formation of TiO_2_-related defect states and SF/TiO_2_ interfacial trap states. These trap states can suppress leakage transport, stabilize the HRS, improve the I_on_/I_off_ ratio, and contribute to interfacial charge storage. Therefore, the combined optical and electrical results consistently support that TiO_2_-induced defect/interface states play a key role in improving the resistive switching and charge storage behavior. To further clarify the performance of the present Ag/SF–TiO_2_/Pt memristor, Table 1 compares the device characteristics with previously reported silk fibroin- and polymer-based memristors. The present device exhibits low-voltage operation, stable endurance and retention, an enhanced memory window, and resistance state-dependent capacitance behavior.

## 4. Conclusions

In this study, Ag/SF–TiO_2_/Pt bio-memristor devices were fabricated using SF–TiO_2_ composite films with different TiO_2_ nanoparticle concentrations. SEM analysis confirmed that TiO_2_ incorporation gradually increased particle aggregation while maintaining continuous film coverage without severe cracks or delamination. Optical analyses revealed that TiO_2_ nanoparticles reduced the apparent optical gap, enhanced sub-bandgap absorption, and suppressed PL intensity, indicating the formation of defect- and trap-related states in the SF matrix. Electrical measurements showed that TiO_2_ incorporation effectively suppressed the initial leakage current and stabilized the high-resistance state. The devices exhibited stable bipolar resistive switching within ±1 V, with enhanced I_on_/I_off_ ratios of approximately 10^4^–10^5^ after TiO_2_ addition. Endurance and retention measurements further confirmed reliable switching over 100 cycles and stable resistance states up to 10^4^ s. Capacitance measurements demonstrated resistance state-dependent charge storage behavior, with significantly enhanced capacitance in the LRS due to conductive filament formation and TiO_2_-assisted interfacial polarization. These results indicate that TiO_2_ nanoparticles play a crucial role in modulating charge trapping, leakage current, resistive switching, and capacitance behavior. Therefore, Ag/SF–TiO_2_/Pt memristors are promising candidates for bio-compatible non-volatile memory and multifunctional memory devices.

## Figures and Tables

**Table 1:** Comparison of the present Ag/SF–TiO_2_/Pt memristor with previously reported SF- and polymer-based memristors. (NR: not reported; DC: direct current).

Device Structure	Active Material	Operating Voltage	Endurance	Retention	Memory Window	Ref.
Ag/SF–Ag NP/Si	Silk fibroin with Ag nanoparticles	V_Set_ ~2.5 V	100 cycles	NR	up to ~10^4^	[1]
Au/SF/Ag, Pt, Cu	SF	~0.8–4.5 V	>30 cycles	>96 h	10^4^–10^6^	[10]
Al/SF/ITO	Natural SF protein	NR	~120 cycles	<900 s	~10	[11]
SF-based biomemristor	Silk fibroin	V_Set_ ~0.7 V	100 cycles	~10^4^ s	~10^3^	[15]
Al/agarose @AuNPs/ITO-PEN	Agarose with Au nanoparticles	V_Set_ ~0.84 V	250 DC cycles	10^5^ s	~10^4^	[16]
Ag/SF–TiO_2_/Pt	SF with TiO_2_ nanoparticles	±1 V V_Set_ ~0.63 V	100 cycles	10^4^ s	10^4^–10^5^	This work

## Data Availability

The data presented in this study are available on request from the corresponding author. The data are not publicly available due to privacy concerns.
